# Associations between soil microbiomes and carbon stabilization under long-term no-till farming systems in the Argentine Pampas

**DOI:** 10.1038/s41598-026-47621-4

**Published:** 2026-04-12

**Authors:** Maximiliano Gortari, Vanina Giselle Maguire, Juan Pedro Ezquiaga, Mariano Cicchino, Matías Bailleres, Roberto Ulises Escaray, Oscar Adolfo Ruiz, María Eugenia Llames

**Affiliations:** 1 Instituto Tecnológico de Chascomús (INTECH), Consejo Nacional de Investigaciones Científicas y Técnicas (CONICET), Av. Intendente Marino km 8.2, Chascomús, Buenos Aires 7130 Argentina; 2 Escuela de Bio y Nanotecnología (EByN), Universidad Nacional de San Martín (UNSAM), Av. Intendente Marino km 8.2,, Chascomús, 7130 Argentina; 3 Estación Experimental Agropecuaria Manfredi, Instituto Nacional de Tecnología Agropecuaria (EEA-INTA), Área Mejoramiento Genético Vegetal, Manfredi, Córdoba 5988 Argentina; 4 Chacra Experimental Chascomús, Ministerio de Desarrollo Agrario (MDA-INTA), Chascomús, Buenos Aires 7130 Argentina; 5 Instituto Nacional de Tecnología Agropecuaria (INTA), EEA Cuenca del Salado, AER, Chascomús, 7130 Argentina

**Keywords:** Soil microbial communities, Total organic carbon stocks, No-till farming, Conventional tillage, Fungal guilds, Agroecosystems, Ecology, Ecology, Environmental sciences, Microbiology

## Abstract

Soil microbial communities play a key role in carbon (C) cycling in agroecosystems; however, their long-term responses to contrasting management practices remain poorly understood in agricultural soils. In this study, we evaluated the effects of more than 20 years of no-till farming (NTF) and conventional tillage (CT) on soil physicochemical properties, bacterial and fungal community composition, and inferred functions related to C and nutrient cycling in the Argentine Pampas. We show that NTF increased total organic carbon (TOC) stocks in surface soils and promoted edaphic conditions associated with C stabilization, including higher cation exchange capacity and structural stability. Bacterial communities exhibited high functional redundancy and were primarily structured along sodium-related parameters, whereas fungal communities were more sensitive to management, with NTF favoring ligninolytic and symbiotic fungi that contribute to necromass formation and long-term carbon stabilization. In contrast, CT enriched opportunistic fungal guilds associated with disturbance and short-term nutrient turnover. Phylogenetic analyses revealed community assembly dominated by environmental filtering in both microbial domains. Overall, these results highlight the central role of fungi as mediators of soil C stabilization and suggest that conservation practices such as NTF enhance microbiome contributions to ecosystem services and climate change mitigation in intensively managed agroecosystems.

## Introduction

Soil, a finite and non-renewable natural resource, is the largest terrestrial reservoir of carbon (C) and provides essential ecosystem functions, including water regulation, biodiversity conservation, and nutrient cycling^1,2^. In agricultural systems, soil C sequestration and stabilization are strongly influenced by management practices that modify soil structure, soil organic matter (SOM) inputs, and the activity of soil microbial communities^3,4^. Consequently, soil management plays a fundamental role in climate change mitigation strategies^5^.

Among agricultural practices, tillage intensity is a key factor that has profoundly affected soil quality, biodiversity, and carbon stocks by accelerating SOM decomposition and reducing C storage^6^. In contrast, conservation-oriented practices such as reduced tillage, residue retention, and diversified crop rotations improve soil structure, support microbial networks, and promote C stabilization^7,8^. These contrasting effects make long-term tillage systems particularly suitable for evaluating the role of soil microbiomes in carbon stabilization.

Soil microorganisms are key drivers of the C cycle, contributing to SOM decomposition and soil organic carbon (SOC) stabilization through biomass turnover and metabolite production^4,9,10^. Land-use changes can leave legacy effects on microbial communities, often resulting in predictable shifts from communities resembling their former state toward those characteristic of the new management regime^11^.

Responses of soil microbiomes to tillage are not uniform across microbial groups. Fungal communities are particularly sensitive to physical disturbances due to the disruption of hyphal networks, often resulting in reduced abundance of symbiotic fungi and a shift toward saprotrophic guilds under conventional tillage (CT)^12,13^. In contrast, bacterial communities generally exhibit relatively greater compositional stability under long-term management, reflecting high functional redundancy and resilience to physical disturbance, although tillage can influence bacterial dispersal and spatial organization in surface soils^14–16^. These patterns indicate that soil microbiomes respond sensitively to management practices and play a crucial role in maintaining soil health and long-term ecosystem functioning, although the strength and direction of these responses are context-dependent and vary with soil type, depth, and cropping system^11,14^.

In the Argentine Pampas, a major temperate grassland and one of the world’s most productive agricultural regions, intensification and monocultures such as soybean and wheat have degraded soil physicochemical and biological properties^17,18^. Continuous cropping without rotation has reduced SOC by approximately 10% in soybean monocultures compared to rotations, with average losses of 8.1 t C ha⁻¹ over ten years and, in some cases, up to 50% losses in specific carbon fractions under minimal rotations^19,20^. Although no-till farming (NTF) with residue retention can improve SOC and preserve soil structure, the effects of these practices on soil microbial structure, composition, and function, and their links to long-term C storage, remain poorly understood^21,22^.

Despite extensive research on SOC dynamics in agroecosystems^4,5^, most studies have focused on individual microbial groups or solely on soil physicochemical parameters^23,24^. Few studies have simultaneously examined multiple microbial communities, limiting our understanding of how the combined composition and dynamics of bacteria and fungi influence soil processes^25–27^. These combined microbial patterns are critical for SOM decomposition, nutrient cycling, and C stabilization and may influence the outcomes of conservation practices such as NTF or residue retention^28,29^. Integrating bacterial and fungal analyses provides a more comprehensive view of ecosystem processes driving C dynamics^21,30–32^, which is essential for identifying sensitive microbial indicators of sustainable soil management and elucidating the mechanisms linking tillage practices to long-term C storage^21^.

Here, we assess the effects of more than 20 years of NTF and CT management on soil physicochemical properties, bacterial and fungal community structure and composition, and inferred functions related to C and nutrient cycling. We also examine phylogenetic structure to infer community assembly processes and propose sensitive microbial indicators to monitor management effects on soil quality and C storage^33^. Additionally, a nearby non-cultivated reference soil was included solely to provide contextual comparisons of soil organic carbon, particulate organic carbon, and total nitrogen stocks, without being considered in hypothesis testing or statistical analyses.

We hypothesize that NTF promotes microbial communities with greater potential functional specialization for C stabilization, resulting in higher C stocks than under CT^34^, with detectable shifts in fungal guilds associated with SOM decomposition and plant–fungal symbioses^30^. In contrast, we expect bacterial communities to exhibit comparatively greater compositional stability under long-term management, reflecting high functional redundancy and resilience to physical disturbances commonly reported for soil bacterial assemblages^15,16^. By integrating microbial ecology and soil science in an intensive agricultural context, this work contributes to understanding how soil microbiomes mediate C storage and ecosystem services relevant to climate change mitigation^35,36^, aligning with the Sustainable Development Goals on climate action, sustainable agriculture, and life on land (SDGs 13, 2, and 15)^37,38^.

## Materials and methods

### Study site

**Fig. 1:**
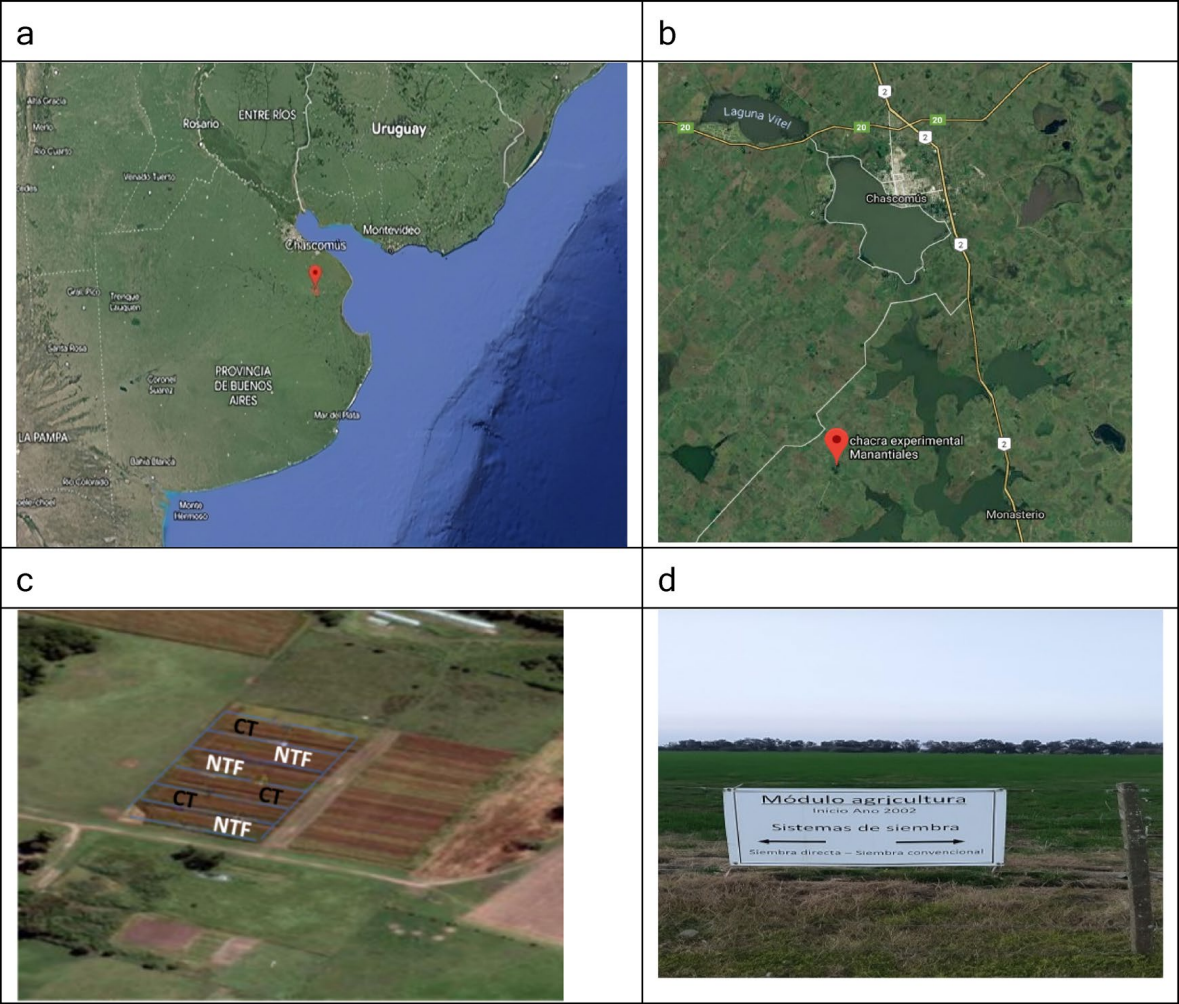
(**a**) Source: Google (n.d.). Chascomús, Buenos Aires, Argentina [Map]. Google Maps. Retrieved September 17, 2025, from (https://www.google.com/maps/place/Chascomús,+Provincia+de+Buenos+Aires). **(b)** Source: Google (n.d.). Chacra Integrated Experimental Chascomús (CEICh) [Map]. Google Maps. Retrieved September 17, 2025, from (https://www.google.com/maps/place/35°44’36.7"S+58°03’25.4"W). (**c**) Source: Google (n.d.). No-Till Farming (NTF) vs. Conventional Tillage (CT) trial. -35°44′38.2″S 58°03′11.1″W [Map]. Google Maps. Retrieved September 17, 2025, from (https://www.google.com/maps/place/35°44’38.2"S+58°03’11.1"W). (**d**) View of the experimental plot. Photograph taken by Maximiliano Gortari on August 25, 2022.

The study was conducted at the Chascomús Experimental Farm (CECh) (35°44’52.44’’ S; 58°02’56.48’’ W)^39^ (Fig. 1a, b), located in the Pampas region of Argentina. The soil under study was classified as a fine, illitic, abrupt, thermic Argiudoll^40^. The regional climate is temperate, with an average annual precipitation ranging from 800 to 1000 mm and a mean annual temperature of 16 °C, with average summer and winter temperatures of 23 °C and 9 °C, respectively.

Soil samples were collected on March 11, 2021, from experimental plots that have been part of a complete randomized block design for over 20 years. The main experimental factor consisted of tillage systems applied to each plot throughout this period. This factor included two levels (each representing a different tillage system), with three replicates per treatment. The experimental units were plots measuring 30 m in width and 50 m in length, randomly assigned to each treatment within each block (Fig. 1c, d): (a) NTF, in which only a narrow strip of soil (0.05 m) was opened for seed placement; (b) CT, involving disk plowing and tine harrowing to a depth of 0.20 m, followed by surface leveling prior to crop sowing.

Throughout the duration of the experiment, each plot—under its assigned tillage system—was subjected to the same annual crop rotation (winter and summer crops), using an identical fertilization plan and consistent applications of herbicides, fungicides, and insecticides until harvest. At the time of sampling, all plots had a standing maize crop (*Zea mays L.*) corresponding to the summer growing season.

In addition to the experimental plots, a nearby non-cultivated reference soil was sampled exclusively to provide contextual comparisons of soil organic carbon, particulate organic carbon, and total nitrogen stocks. This reference soil was not included in the experimental design, nor was it considered in statistical analyses or hypothesis testing.

### Sample collection

In each experimental unit (i.e., plot), three composite bulk soil samples were collected at two depths: 0–10 cm and 10–30 cm. Sampling was conducted using a soil auger within a radius of 5 to 10 m, targeting soil at least 10 cm away from visible maize roots; rhizosphere soil was not intentionally included. The samples were placed in plastic bags, homogenized, and properly labeled for subsequent analysis.

### Determination of soil physicochemical parameters

Soil samples intended for physicochemical analysis were air-dried, gently disaggregated, and sieved (2 mm) to ensure homogenization, excluding visible fine roots that could distort measurements of stable organic carbon and nitrogen content. The presence of reactive mineral carbonates was assessed prior to determining total C and nitrogen (N).

Bulk density (BD) was determined using the field core method. Soil texture and chemical parameters were analyzed by a laboratory affiliated with the SAMLA (SUELO ARGENTINO – Comprehensive Soil Analysis Laboratory), following IRAM-SAGyP standards^41^.

The calculation of total organic carbon (TOC), particulate organic carbon (POC), and total nitrogen (TN) stocks (expressed in megagrams per hectare) was performed using the formula proposed by^42^, which considers concentration, horizon thickness, and bulk density:

$$\:{S}{t}{o}{c}{k}\:\left({M}{g}{\:{h}{a}}^{-1}\right)=\left(\frac{{x}}{100}\right){*}{B}{D}{*}{p}{*}{10}^{4\:}{{m}}^{2\:\:}{{h}{a}}^{-1}$$

Where BD is bulk density (Mg m⁻³), p is soil layer thickness (in meters), and x is the percentage content of TOC, POC, or TN (or their respective fractions).

Physical parameters such as volumetric water content (WCON, m³/m³), temperature (TEMP, °C), and electrical conductivity (EC, dS/m) were measured at 0–10 cm and 10–30 cm soil depths using a PROCHECK reader equipped with METER GS3 sensors. Soil penetration resistance (SPR, kPa) was measured along the entire 0–30 cm soil profile using a handheld digital cone penetrometer (FieldScout SC-900, Spectrum Technologies Inc.).

The chemical parameters determined included TOC (%), POC (%), TN (%), nitrate nitrogen (N_NO₃, ppm), ammonium nitrogen (N–NH₄, ppm), and the C/N ratio. These were analyzed by mass spectrometry using a LECO TruSpec CN analyzer at the Soil, Plant and Environment Analytical Services Department (LABSPA) of CERZOS (CONICET). Additional measurements included pH (1:2.5), EC, available phosphorus (P, Bray I), soluble and exchangeable anions and cations, sodium adsorption ratio (SAR), cation exchange capacity (CEC), percentage of exchangeable sodium (ESP), saturation moisture content (%, MC), and particle-size fractions (%) of silt, clay, and sand.

### DNA extraction and sequencing (illumina MiSeq)

Samples intended for the study of microbial communities (i.e., bacterial and fungal) were transported to the laboratory and stored at 4 °C until analysis. DNA was extracted from 0.25 g of soil using the Qiagen^®^ DNeasy PowerSoil Kit, following the standard protocol. DNA concentration and quality were assessed via spectrophotometry (Synergy™ H1, BIOTEK) and 1.5% agarose gel electrophoresis.

For the construction of both bacterial and fungal libraries, 10 ng of DNA was used as template in PCR reactions. The final libraries consisted of double-stranded DNA fragments approximately 600 base pairs in length. To amplify the hypervariable V3–V4 region of the 16 S rRNA gene, primers MiSeq341F (5′–3′: ACACTCTTTCCCTACACGAC) and MiSeq805R (5′–3′: GTGACTGGAGTTCAGACGTG) were used^43^. For amplification of the ITS2 region, primers ITS3-F (5′–3′: GCATCGATGAAGAACGCAGC) and ITS4-R (5′–3′: TCCTCCGCTTATTGATATGC) were used^45^. These primer sets are widely validated for bacterial and fungal community profiling in agricultural soils. Although no soil-specific preliminary primer coverage test was conducted, sequencing quality and taxonomic coverage were evaluated posteriori through stringent quality filtering, DADA2 (version 1.22.0) based amplicon sequence variants (ASV) inference, removal of low-abundance and non-target sequences, and the taxonomic resolution obtained using the SILVA (version 138) and UNITE (version 8.3) reference databases.

The PCR protocol used was as follows (final volume: 25 µL): 95 °C for 3 min, followed by 25 cycles of 95 °C for 30 s, 55 °C for 30 s, and 72 °C for 30 s. This was followed by a final extension at 72 °C for 5 min, and a hold at 4 °C. Both amplicon library preparation and sequencing of bacterial and fungal communities were performed using the Illumina MiSeq platform with a 2 × 300 bp paired-end format, suitable for microbial community structure studies^45^. Amplicon libraries and sequencing were carried out at the Genome Quebec Innovation Centre (Montreal, Canada).

### Bioinformatic analysis of sequences

Sequence preprocessing was carried out using QIIME 2 (Quantitative Insights Into Microbial Ecology 2, version 2022.11), developed by^46^. The initial step consisted of raw sequence quality control using FastQC (version 0.11.9) in a Linux environment. After primer removal and quality filtering, the assignment of ASVs was performed using the DADA2 pipeline^47^.

Taxonomic assignment was performed using the SILVA database for bacteria^48^ and the UNITE database for fungi^49^. Classification was carried out using the Naive Bayes classifier and the q2-feature-classifier plugin within QIIME 2. Sequence alignment was conducted using MAFFT (version 7.505)^50^, and phylogenetic trees were constructed using the FastTree algorithm^51^ in a Linux environment.

### Statistical analyses

Statistical differences in soil carbon and nitrogen stocks (TOC, POC, and TN) between tillage systems (NTF and CT) were evaluated using one-way analysis of variance (ANOVA). When significant effects were detected, mean separation was performed using Tukey’s HSD test with a significance level of α = 0.05. These analyses were applied exclusively to the experimental treatments. The reference soil was used only for contextual comparison of stock values and was not included in the statistical analyses.

For the statistical analysis of bacterial and fungal microbiomes, sequences with a single read (singletons), those assigned to chloroplasts or mitochondria, and sequences with taxonomic assignments outside the targeted groups were removed, as they do not provide relevant information. After this initial filtering, the resulting ASV matrix was further refined by retaining only those ASVs with a total read count exceeding 0.01% of the dataset. All analyses were conducted using the open-source software R^52^. The outputs generated in QIIME 2, including ASV abundance tables for both microbial communities, taxonomic assignment tables, representative sequences, phylogenetic trees, and metadata tables, were imported into R using the *qiime2R* package.

#### Soil physicochemical analyses

To describe the physical and chemical characteristics of the soils and evaluate edaphic patterns associated with the management systems, a Principal Component Analysis (PCA) was performed using the *PCA ()* function from the *FactoMineR* package in R. Prior to analysis, the data were standardized using the *decostand ()* function. Variables with the highest contributions to the first two principal components were identified using the *fviz_contrib ()* function from *FactoMineR*. Highly collinear variables (VIF > 0.65) were detected and removed using the *vifcor ()* function. Subsequently, a PERMANOVA was conducted on the PCA pattern using the *adonis2()* function from the *vegan* package, and the homogeneity of multivariate dispersions was tested with *betadisp ()* (also from *vegan*) to assess the robustness of the analysis^53^.

#### Alpha diversity

Alpha diversity indices were calculated using the *estimate_richness ()* function from the *phyloseq* package. The indices included: observed richness as the number of ASVs present^54^; Chao1, which estimates richness by relating expected to observed ASVs; ACE (abundance-based coverage estimator); Shannon entropy; Simpson dominance, which evaluates the probability that two individuals randomly selected belong to different species; Inverse Simpson; Fisher’s alpha; and Phylogenetic Diversity (PD)^55^. To assess statistical differences between tillage systems, Kruskal–Wallis tests were applied to each of the aforementioned diversity indices, using a significance level of 0.05.

Through the analysis of these alpha diversity indices, it is possible to characterize both the species richness of each microbial community and its structure, identifying ASVs that may dominate the community as well as those occurring at low relative abundance.

#### Microbial community composition

Beta diversity analysis allows for the assessment of compositional turnover between microbial communities and quantifies their (dis)similarities. In this study, a Bray–Curtis distance matrix was computed using the *ordinate ()* function from the *phyloseq* package. This matrix was used to generate an ordination of the experimental units based on community composition, applying the non-metric multidimensional scaling (NMDS) technique, which enables the representation of complex ecological relationships in a reduced dimensional space.

The homogeneity of multivariate dispersions among treatments was assessed using the *betadisper ()* function from the *vegan* package, in order to validate the assumptions required for the application of permutational analysis of variance (PERMANOVA). Subsequently, a PERMANOVA was performed using the *adonis2()* function, with 999 permutations, to evaluate the statistical significance of the differences observed in the NMDS ordination space.

To explore the influence of environmental variables on microbial community structure, environmental vectors were fitted to the NMDS ordination space using the *envfit ()* function from the vegan package, with 999 permutations. Prior to this, collinearity among correlated variables (*p* < 0.05) was assessed to avoid redundancy in the interpretation. The selected variables were incorporated as explanatory covariates in differential abundance models using ANCOM-BC, with the aim of identifying edaphic factors associated with compositional changes, with particular interest in those potentially modulating soil carbon stabilization capacity.

For microbial community analyses, a total of six samples per tillage system were included (three per depth). Given the limited number of samples per depth, soil depth was not analyzed as a categorical factor but evaluated as a continuous environmental variable using vector fitting.

#### Differential abundance analysis

The *ANCOM-BC* package (version 2.8.1;^56^) (Analysis of Composition of Microbiomes with Bias Correction) was used to identify phyla, classes, and ASVs that exhibited differential abundance between tillage systems. This approach detects taxa whose mean proportions differ significantly between experimental groups^57^. The analysis was performed using default parameters, except for p-value adjustment, which was applied using the Benjamini–Hochberg method to control the false discovery rate (FDR), with statistical significance defined as adjusted p-values (FDR) < 0.05^56^, and the option neg_lb = TRUE was enabled. Explanatory variables were standardized beforehand to avoid scale-related bias. Differential abundance analysis was only conducted when significant differences were observed between management systems in the NMDS ordination.

Log fold change (LFC) values reported throughout the manuscript correspond to log2-transformed fold changes, as implemented by default in the *ANCOM-BC* framework.

#### Microbial functional prediction

Bacterial functional predictions were generated based on 16 S rRNA gene data using the *Tax4Fun2* package^58^, referencing the extended “Ref100” database. Pathway abundances were normalized according to the 16 S rRNA gene copy number and used as input for functional profiling. Predicted functions were grouped according to metabolic pathways defined by the Kyoto Encyclopedia of Genes and Genomes (KEGG). These predictions represent potential functional capacities inferred from taxonomic profiles and do not reflect actual gene expression levels or in situ metabolic activity.

For fungi, the *FUNGuild* package^59^ was used to assign functional guilds based on taxonomic classifications derived from ITS2 ASVs, enabling the characterization of ecological roles within communities under both management systems. Guild assignments were interpreted as functional tendencies rather than direct measures of fungal activity.

The functional matrices derived from both approaches were integrated into phyloseq *objects* and used to compute NMDS ordinations based on Bray–Curtis distances, in order to evaluate the functional structure of microbial communities and explore potential functional implications related to nutrient cycling and soil carbon accumulation. As in the taxonomic analysis, PERMANOVA was applied following an assessment of dispersion homogeneity, and environmental vectors were fitted to the NMDS space using the *envfit ()* function when significant differences between treatments were detected.

Differential analysis using ANCOM-BC was performed only when significant functional differences were observed in the NMDS ordination between tillage systems. The statistical model included both the management type and the significant environmental variables identified. Collinearity among variables was assessed prior to inclusion in the model, and redundant variables were removed. The analysis was conducted at both the level of grouped functions and individual ASVs with functional assignments.

#### Phylogenetic structure analysis

To assess the organization of microbial communities in relation to their evolutionary history and to explore the ecological processes shaping them, we calculated phylogenetic distance-based indices (SES.MNTD and SES.MPD). These metrics allow the detection of phylogenetic clustering or overdispersion. Values obtained for each community were compared between tillage systems, and their statistical significance was evaluated through permutations. The methodology follows the approach described in^60^, where full technical details are provided.

## Results

### Soil carbon and nitrogen stocks under contrasting tillage systems and reference soil

**Fig. 2:**
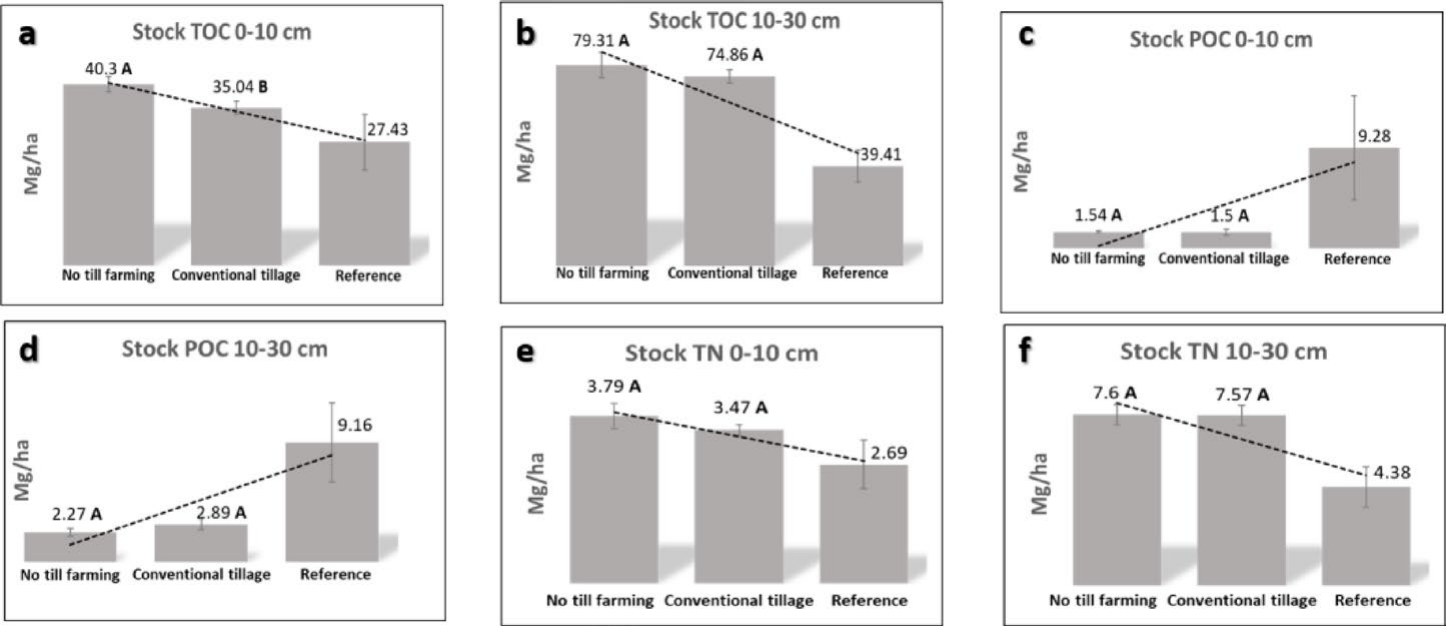
(**a**) Mean values of TOC stock from 3 replicates at 0–10 cm depth under NTF and CT, and from 10 replicates for the reference system. (**b**) Mean values of TOC stock from 3 replicates at 10–30 cm depth under NTF and CT, and from 10 replicates for the reference system. (**c**) Mean values of POC stock from 3 replicates at 0–10 cm depth under NTF and CT, and from 10 replicates for the reference system. (**d**) Mean values of POC stock from 3 replicates at 10–30 cm depth under NTF and CT, and from 10 replicates for the reference system. (**e**) Mean values of TN stock from 3 replicates at 0–10 cm depth under NTF and CT, and from 10 replicates for the reference system. (**f**) Mean values of TN stock from 3 replicates at 10–30 cm depth under NTF and CT, and from 10 replicates for the reference system. Statistical analysis was performed using ANOVA (Tukey, α = 0.05). Mean values between NTF and CT were compared. Different letters indicate significant differences.

In Fig. 2, the values obtained for the parameters related to C and N under both soil management practices, at the two considered depths, are shown and compared against the reference soil (reference values ​​obtained in 2019 from grassland plots within CECh). In Fig. 2a, it can be observed that the TOC stock at 0–10 cm was higher (p-value = 0.02) under NTF compared to the TOC stock in the CT system, and in both cases, the values were greater than those obtained for the reference soil. At 10–30 cm, no differences (p-value = 0.26) were found in TOC stock between tillage systems; however, in both depths, the values of the tillage systems were higher than those of the reference soil (Fig. 2b).

As shown in Fig. 2c and d, no differences (p-value = 0.86 at 0–10 cm; p-value = 0.13 at 10–30 cm) were found in POC stock between tillage systems at either depth. When comparing POC stocks of the tillage systems with those of the reference soil, in this case, at both depths, the values were higher in the latter system.

Regarding TN stock, no differences were found (p-value = 0.18 at 0–10 cm; p-value = 0.93 at 10–30 cm) between tillage systems at either depth (Fig. 2e and f). Compared to the values of the reference soil, both under NTF and CT and at both depths, TN stocks were higher in the tillage systems.

### Multivariate analysis of soil physicochemical properties under contrasting tillage systems

**Fig. 3:**
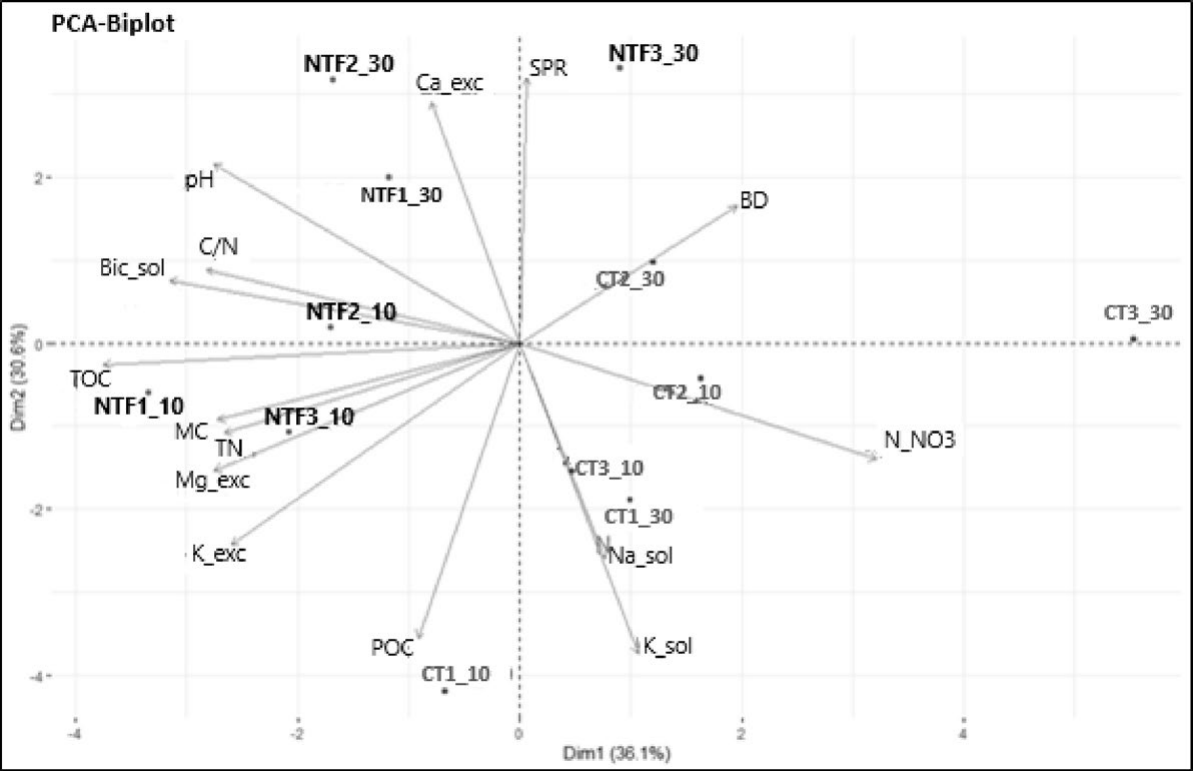
PCA of sampling sites and depths based on environmental variables. No-till farming (NTF1_10, NTF1_30, NTF2_10, NTF2_30, NTF3_10, NTF3_30) and conventional tillage (CT1_10, CT1_30, CT2_10, CT2_30, CT3_10, CT3_30). TOC: Total organic carbon, POC: Particulate organic carbon, TN: Total nitrogen, N_NO3: Nitrate, C/N: Carbon-to-nitrogen ratio, BD: Bulk density, SPR: Penetration resistance, K_exc: Exchangeable potassium, Mg_exc: Exchangeable magnesium, Ca_exc: Exchangeable calcium, Na_sol: Soluble sodium, K_sol: Soluble potassium, Bic_sol: Soluble bicarbonate, pH: Hydrogen potential, MC: Moisture Content.

Figure 3 shows the ordination obtained from the PCA. Dimension 1 (Dim 1) explained the highest proportion of variability (36.1%), and together with Dimension 2 (Dim 2), accounted for 66.7% of the total variability observed. The first component (Dim 1) correlated positively with N_NO₃, K_sol (soluble potassium), Na_sol (soluble sodium), BD, and POC, and negatively with pH, TOC, C/N, Bic_sol (soluble bicarbonate), Ca_exc (exchangeable calcium), SPR, TN, MC, Mg_exc (exchangeable magnesium), and K_exc (exchangeable potassium), indicating a gradient of exchangeable macronutrients such as Ca_exc, Mg_exc, and K_exc, which are part of the CEC. On the other hand, Dim 2 correlated positively with POC, K_exc, Mg_exc, TN, MC, K_sol, TOC, Na_sol, Bic_sol, and C/N, and negatively with SPR, BD, Ca_exc, N_NO₃, and pH, suggesting a gradient of nutrient availability, including soluble anions and cations such as K_sol, Na_sol, and Bic_sol. The physicochemical conditions of soils under different tillage systems differed significantly (PERMANOVA, *p* = 0.004). Overall, soils under NTF were more associated with higher CEC, pH, higher C/N, TOC, MC, TN, Bic_sol, and SPR compared to CT soils. Conversely, CT soils were more associated with higher BD, N_NO₃, K_sol, Na_sol, and POC than NTF soils.

The complete set of physicochemical parameters for both tillage systems is provided in the Supplementary Table S1, which allows a more detailed comparison of the measured variables across treatments.

### Alpha and beta diversity patterns of bacterial and fungal communities under contrasting tillage systems

In general, no significant differences were observed in the alpha diversity indices between the NTF and CT systems (*p* > 0.05; Table S2). Both systems exhibited high species richness, accompanied by the dominance of a few ASVs. As shown in Table S2, none of the diversity indices (Observed, Chao1, ACE, Shannon, Simpson, InvSimpson, Fisher, and PD) revealed statistical differences between the compared systems.

**Fig. 4:**
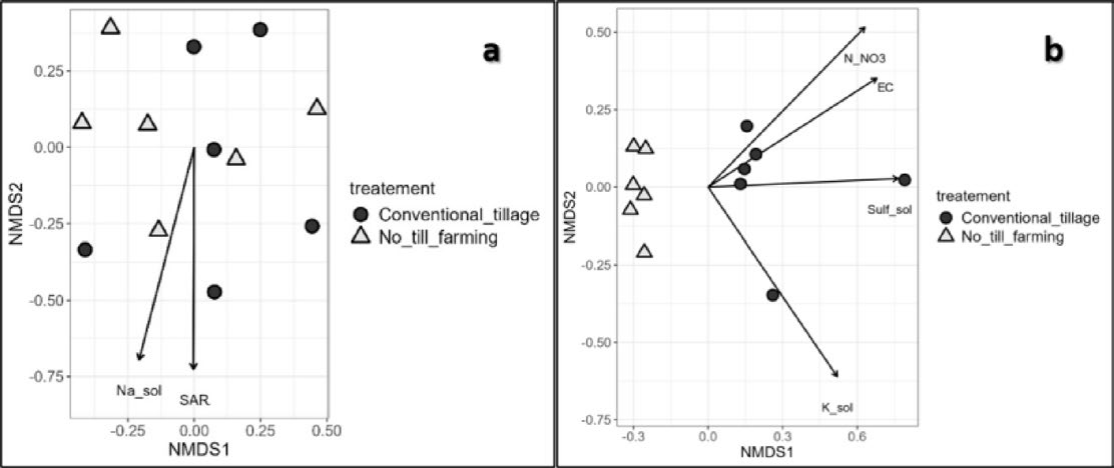
Non-metric multidimensional scaling (NMDS) ordination of soil microbial communities under no-till farming (NTF) and conventional tillage (CT). (**a**) Bacterial community composition with the environmental variables that were most relevant in the ordination (Na_sol and SAR). Groups did not differ significantly between tillage systems (PERMANOVA, *p* = 0.86) (stress = 0.095). (**b**) Fungal community composition showing significant differences between tillage systems (PERMANOVA, *p* = 0.003). Environmental variables significantly correlated with the ordination were K_sol, Sulf_sol, EC and N_NO3 (stress = 0.062).

The beta diversity analysis indicates that, for the bacterial community, the groups observed in the overall data pattern, described through NMDS ordination, did not differ significantly between tillage systems (PERMANOVA, p-value = 0.86). Soil depth was not included as a main or random factor in the PERMANOVA model and was evaluated separately as an environmental variable. The homogeneity of multivariate dispersions between tillage systems was confirmed (betadisper, *p* > 0.05), indicating that the PERMANOVA result reflects differences in centroid location rather than dispersion effects. Despite the absence of significant differences, the ordination plot was constructed considering the environmental variables that were most relevant for selection, which in this case were Na_sol and SAR (Fig. 4a, Table S3). These variables were the most strongly correlated with the observed distribution, indicating that edaphic conditions associated with sodium are structuring the communities along the observed edaphic parameters.

Regarding the fungal community, the groups observed in the overall data pattern, described through NMDS ordination, differed significantly between tillage systems (PERMANOVA, p-value = 0.003). Soil depth was not included as a main or random factor in the PERMANOVA model and was evaluated separately as an environmental variable. Homogeneity of multivariate dispersions between tillage systems was confirmed (betadisper, *p* > 0.05). The ordination plot was constructed considering the environmental variables that were significant in the observed pattern, namely K_sol, Sulf_sol (soluble sulfates), EC, and N_NO3 (Fig. 4b, Table S4). Fungal communities under CT appeared to be more strongly influenced by these edaphic parameters.

### Bacterial dominance and differential shifts in fungal communities under contrasting tillage systems

**Fig. 5:**
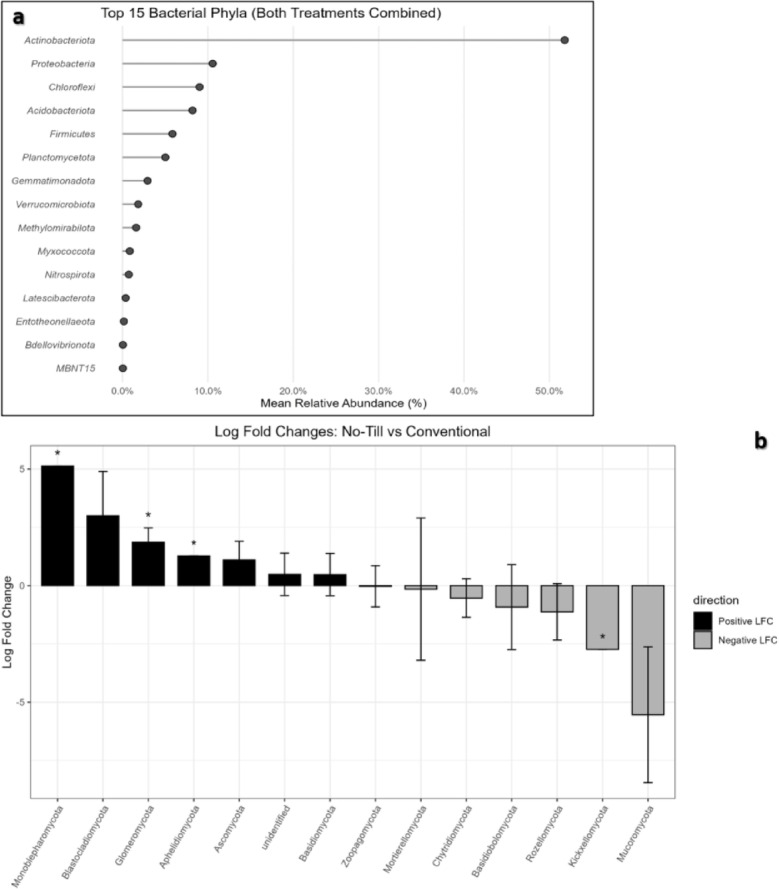
(**a**) Relative abundance of the main bacterial phyla under NTF and CT. (**b**) Differential abundance analysis of fungal phyla between tillage systems. Positive log fold change (LFC) values indicate higher relative abundance under NTF, while negative values indicate higher relative abundance under CT.

Given the absence of significant differences in the bacterial community structure between systems, the taxonomic composition was analyzed considering both systems together. In these soils, the main bacterial *phyla* identified were *Actinobacteriota* (51.8%), *Proteobacteria* (10.6%), *Chloroflexi* (9.03%), *Acidobacteriota* (8.20%), and *Firmicutes* (5.85%) (Fig. 5a).

Regarding fungal communities, inspection of relative abundance profiles across samples showed that *Basidiomycota* and *Ascomycota* overwhelmingly dominated the fungal assemblages in both tillage systems, jointly accounting for more than 90% of total sequences (Supplementary Fig. S2). The remaining fungal community was composed of a diverse set of low-abundance phyla, including *Chytridiomycota*,* Zoopagomycota*, *Rozellomycota*,* Mucoromycota*, and *Glomeromycota*, each representing less than 3% of the total relative abundance. Although *p__unidentified* ranked among the most abundant categories, it was not discussed further due to the lack of taxonomic resolution at the phylum level. Within this compositional context, differential abundance analysis at the phylum level revealed a significant increase in *Monoblepharomycota* (log fold change [LFC] = 5.13, adjusted *p* < 0.001), *Glomeromycota* (LFC = 1.86 ± 0.61, adjusted *p* = 0.023), and *Aphelidiomycota* (LFC = 1.27, adjusted *p* < 0.001) under NTF, along with a significant decrease in *Kickxellomycota* (LFC = − 2.73, adjusted *p* < 0.001) in this system (Fig. 5b).

**Fig. 6:**
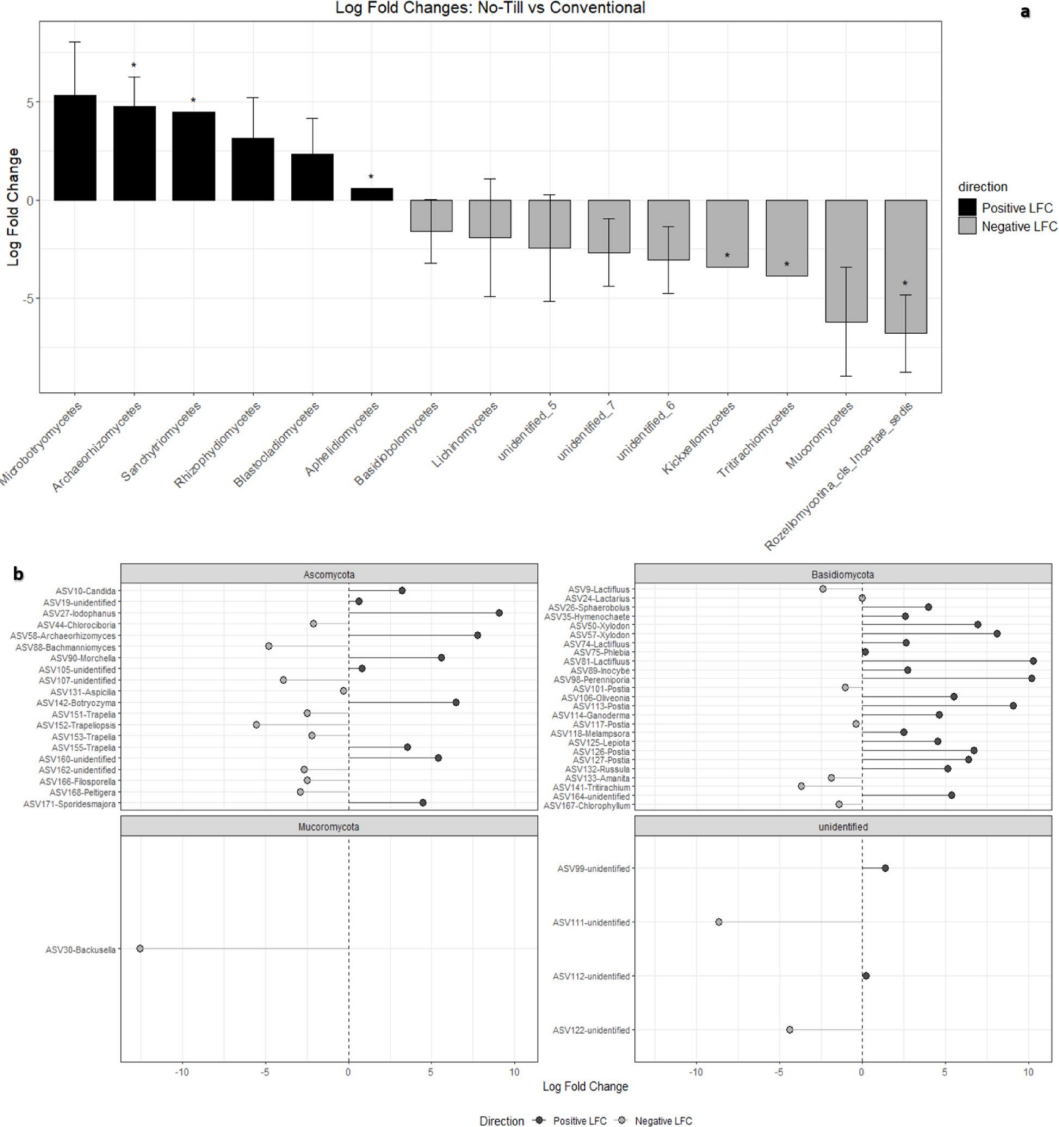
(**a**) Differential abundance analysis of fungal classes between NTF and CT systems. Positive log fold change (LFC) values indicate higher relative abundance under NTF, while negative values indicate higher relative abundance under CT. (**b**) Differential abundance analysis at the ASV level for the 50 most abundant fungal ASVs between NTF and CT systems. Positive log fold change (LFC) values represent ASVs enriched under NTF, while negative values represent ASVs enriched under CT.

At the fungal class level, *Archaeorhizomycetes* ([LFC] = 4.75 ± 1.52, adjusted *p* = 0.039), *Sanchytriomycetes* ([LFC] = 4.46, adjusted *p* < 0.001), and *Aphelidiomycetes* ([LFC] = 0.60, adjusted *p* < 0.001) showed a significant increase under NTF, whereas *c__Rozellomycotina_cls_Incertae_sedis* ([LFC] = − 6.80 ± 1.95, adjusted *p* = 0.022), *Tritirachiomycetes* ([LFC] = − 3.88, adjusted *p* < 0.001), and *Kickxellomycetes* (LFC] = − 3.40, adjusted *p* < 0.001) exhibited a significant decrease in this system (Fig. 6a).

At the ASV level, we identified 240 abundant taxa that exhibited significant differences in relative abundance between tillage systems; for visualization, the 50 most abundant ASVs were considered. Most of them belonged to the *Ascomycota* and *Basidiomycota phyla*. The highest positive LFC values, indicating a significant increase in relative abundance under NTF, were observed in members of the *Basidiomycota phylum* (Fig. 6b).

### Functional profiles of bacterial pathways and fungal guilds under contrasting tillage systems

The main bacterial functional pathways, in terms of relative abundance, were identified using the Tax4Fun2 functional prediction approach. The analysis focused on level 1 pathways related to the C and N cycles. Since no significant differences were observed in the bacterial community structure between tillage systems, the functional pathways were represented by combining data from both systems.

**Fig. 7:**
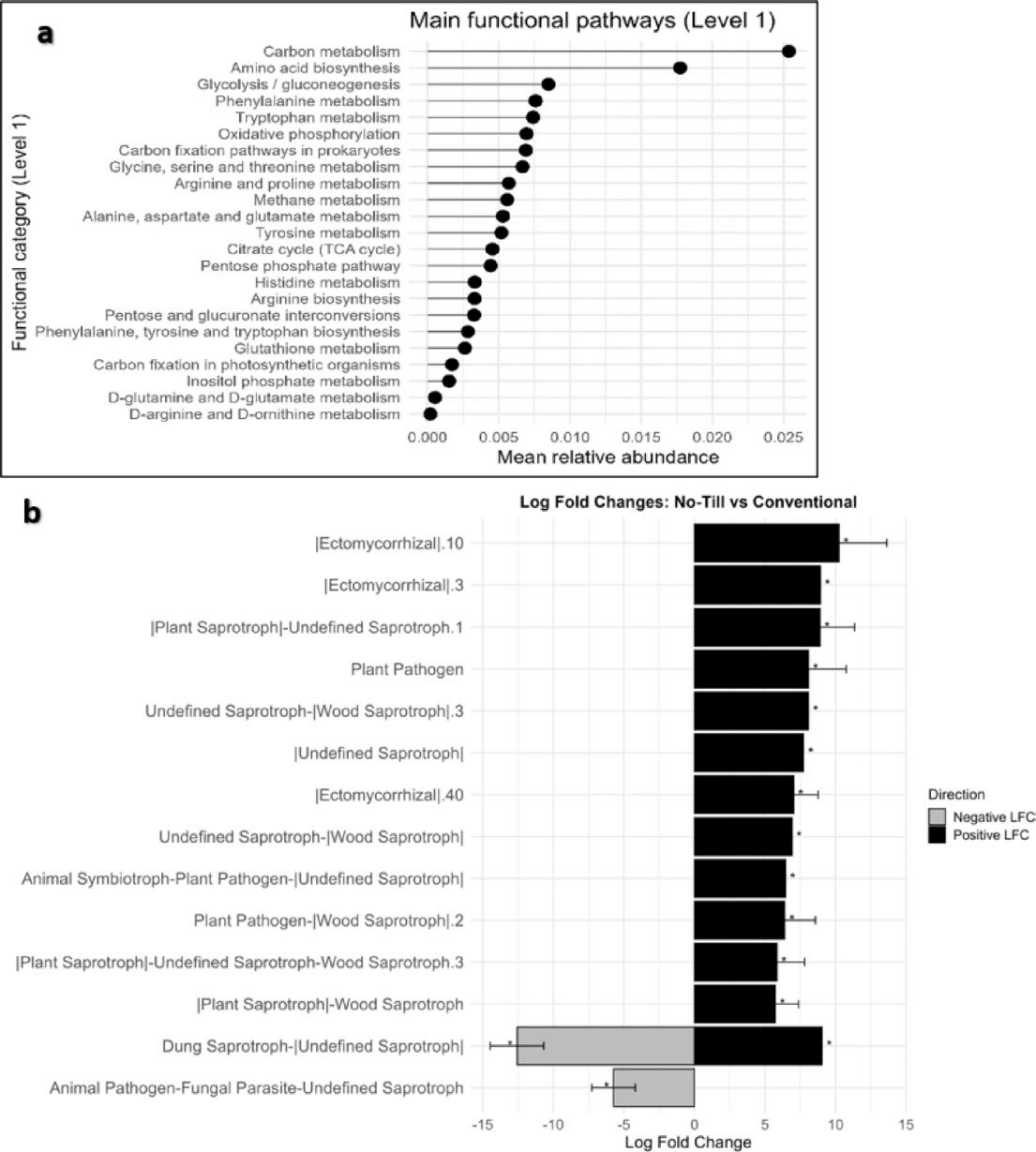
(**a**) Predicted bacterial functional pathways related to C and N cycles identified using the Tax4Fun2 functional prediction tool. Since no significant differences in bacterial community structure were observed between tillage systems, data from both systems were pooled. (**b**) Differential abundance of fungal guilds between tillage systems (stress = 0.082). Positive log fold change (LFC) values indicate higher relative abundance under NTF, while negative values indicate higher relative abundance under CT.

The level 1 functional pathways identified were: Carbon metabolism (0.025%), Amino acid biosynthesis (0.017%), Glycolysis/gluconeogenesis (0.008%), Phenylalanine metabolism (0.0075%), and Tryptophan metabolism (0.0074%) (Fig. 7a).

Regarding the fungal community, the general pattern revealed by NMDS showed significant differences between tillage systems (PERMANOVA, *p* = 0.003). To interpret these differences, environmental variables that were significantly associated with the observed distribution were incorporated into the ordination plot. These variables were: SPR, Sulf_sol, K_sol, EC, and N_NO_3_ (Supplementary Fig. S1, Table S4). Fungal guilds under CT were primarily structured by nutrient-related parameters (K_sol, Sulf_sol, and N-NO₃) and EC, whereas under NTF, guild distribution was more strongly associated with SPR.

Figure 7b shows fungal guilds with significant differences between tillage systems. Guilds such as *Ectomycorrhizal.10*,* Ectomycorrhizal.3*,* Plant Saprotroph–Undefined Saprotroph.1*,* Plant Pathogen*,* Undefined Saprotroph–Wood Saprotroph.3*,* Undefined Saprotroph*,* Ectomycorrhizal.40*,* Undefined Saprotroph–Wood Saprotroph*,* Animal Symbiotroph–Plant Pathogen–Undefined Saprotroph*,* Plant Pathogen–Wood Saprotroph.2*,* Plant Saprotroph–Undefined Saprotroph–Wood Saprotroph.3*,* and Plant Saprotroph–Wood Saprotroph* showed higher relative abundance under NTF.

Guild labels followed by numeric suffixes (e.g., *Ectomycorrhizal.1*, *Ectomycorrhizal.10*) correspond to distinct ASVs sharing the same functional assignment but differing in their taxonomic identity.

Additionally, guilds with the same functional assignment but different taxonomic affiliations were identified; for example, *Dung Saprotroph–Undefined Saprotroph* showed significant differences in both systems due to their shared functional role but distinct taxonomic origins. On the other hand, the guild *Animal Pathogen–Fungal Parasite–Undefined Saprotroph* was more abundant under CT.

### Phylogenetic clustering patterns in bacterial and fungal communities across tillage systems

**Fig. 8:**
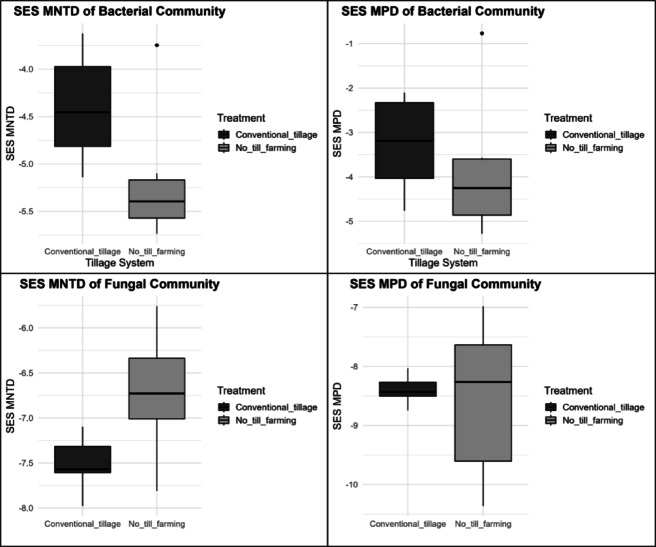
SES MPD and SES MNTD for bacterial and fungal communities under NTF and CT. Both metrics were significantly lower than zero (*p* = 0.001).

The standardized values of Mean Pairwise Distance (SES MPD) and Mean Nearest Taxon Distance (SES MNTD) were significantly lower than zero (*p* = 0.001) for both bacterial and fungal communities across both tillage systems (Fig. 8). These results indicate a non-random community assembly structure, consistent with a pattern of phylogenetic clustering, regardless of the management system.

## Discussion

Our results demonstrate that, after more than 20 years of contrasting management, NTF promoted higher TOC stocks in the upper 10 cm of soil compared to CT (Fig. 2a). This finding is consistent with previous studies showing that reduced physical disturbance and surface residue retention favor C accumulation in surface horizons^61,62^. In contrast, POC and TN stocks did not differ significantly between management systems (Figs. 2c–f), suggesting that the effects of NTF on these fractions may require longer time scales or depend on interactions with crop rotations and residue quality^4,63^. The absence of significant differences in TOC stocks at 10–30 cm between tillage systems, despite the greater soil disturbance under conventional tillage, likely reflects vertical redistribution of surface carbon due to plowing, partially offsetting surface carbon losses at depth rather than indicating enhanced carbon stabilization^61,62^.

In our study, TOC and TN stocks were higher in agricultural soils than in the reference site (native grassland soils), while POC stocks were greater in the latter (Figs. 2a–f). This indicates that intensive agricultural use enriches the surface profile in total C and N but reduces more labile fractions such as POC, in agreement with studies highlighting the sensitivity of these pools to management and their implications for long-term stability^4,64^. Similarly,^65^ emphasize that land use (forest, grassland, cropland) strongly conditions the potential for carbon sequestration, with natural systems showing the highest capacity. Therefore, conservation practices such as NTF represent a management opportunity to move agroecosystems closer to stable C capture, provided they are maintained continuously over time, as in our two-decade experiment.

The edaphic differences revealed by the PCA (Fig. 3) and described in Table S1 highlight that tillage systems shape distinct soil conditions, which in turn steer soil C toward contrasting pathways of stabilization or mineralization. NTF was associated with higher pH, CEC, C/N, TOC, and SPR, conditions linked to C stabilization and resilient fertility^61^. In contrast, CT was associated with higher BD, accumulation of N_NO3, and greater concentrations of K_sol and Na_sol. This agrees with evidence showing that frequent tillage reduces SOM stability by disrupting aggregates and accelerating mineralization^6,66^. In this context, the higher TOC and C/N ratios observed under NTF can be interpreted as a shift toward microbial communities potentially adapted to a more conservative and stable carbon turnover, rather than as direct evidence of increased microbial carbon use efficiency (CUE). CUE is a physiological parameter that cannot be directly inferred from taxonomic data and is therefore discussed here within a conceptual framework. In line with this interpretation,^67^ emphasizes that reduced-disturbance practices tend to favor microbial communities that channel carbon into biomass and necromass rather than rapid respiratory losses, contributing to more persistent soil C pools.

In terms of microbial diversity, no significant differences were observed in alpha diversity indices between NTF and CT (Table S2). Both systems exhibited high species richness, dominated by a few abundant ASVs. This pattern suggests that although management strongly influences community composition, richness and evenness remain stable, in line with studies showing that agricultural practices often alter microbial structure without necessarily reducing alpha diversity^25^. In agricultural soils, management more frequently affects beta diversity than alpha diversity, particularly in fungi, which respond strongly to nutrient availability and disturbance regimes^68,69^. Similarly, land-use change and seasonal variation influence microbial structure and functionality without necessarily altering richness metrics^70^.

At the community level, bacterial structure was relatively stable across management systems (Fig. 4a), whereas fungi responded with greater sensitivity to soil conditions, particularly soluble nutrients and EC (Fig. 4b). Bacterial resilience may be related to their high functional redundancy, but also to the long time elapsed since the last tillage event, which may have allowed community recovery under both management systems^23,24^, while fungi, which rely on recalcitrant resources and symbiotic interactions, are more vulnerable to management-induced changes^68,69^. Our results are consistent, in part, with recent findings showing that fungal diversity responds more strongly to the combined effects of herbicides and tillage in conservation agriculture, with implications for both biodiversity and productivity^71^. These patterns also align with^72^, who demonstrated in mountain grasslands that management practices (fertilization, mowing, grazing, abandonment) markedly alter microbial activity, generating persistent microbial legacies. In our case, two decades under NTF likely consolidated a more stable fungal structure, whereas CT perpetuates a legacy of opportunistic communities associated with rapid mineralization.

Despite the absence of significant differences in bacterial beta diversity between systems, NMDS ordination revealed that bacterial communities were structured along sodium-related parameters (Na_sol, SAR) (Fig. 4a). The dominant bacterial phyla were *Actinobacteriota*,* Proteobacteria*,* Chloroflexi*,* Acidobacteriota*, and *Firmicutes* (Fig. 5a), consistent with previous reports of their prevalence in agroecosystems^23^. Similarly,^73^ found that bacterial communities in South Korean agricultural soils were strongly determined by edaphic factors (pH, nutrients, salinity) rather than geographic location, confirming that soil chemistry regulates microbiome structure. In contrast, fungal beta diversity differed significantly between NTF and CT, with CT communities more strongly influenced by K_sol, Sulf_sol, N_NO₃, and EC. This highlights that bacterial communities are primarily structured by broad edaphic parameters, whereas fungi respond more directly to management systems.

Although no significant differences were detected in the dominant fungal phyla (*Ascomycota*,* Basidiomycota*; Fig. 5b), finer taxonomic levels revealed clear shifts (Fig. 6b). Under NTF, we observed enrichment of ligninolytic and saprotrophic genera such as *Ganoderma*,* Perenniporia*,* Phlebia*, and *Xylodon*, as well as yeasts and root-associated fungi such as *Candida* and *Archaeorhizomyces* (Figs. 5 and 6). These taxa are linked to the decomposition of complex substrates and the generation of fungal necromass, a key component of C stabilization within soil aggregates^10,29,74^. In contrast, CT enriched for opportunistic and disturbance-tolerant taxa such as *Aspicilia*,* Peltigera*,* Tritirachium*, and *Chlorophyllum*, reflecting communities adapted to unstable environments^34,75^. These findings support the idea that taxonomic resolution strongly influences the interpretation of functional processes^76^. Similarly,^77^ showed that NTF in Chinese agroecosystems promoted greater microbial biomass and mycorrhizal associations, with community composition varying substantially with depth, confirming that management effects are most pronounced in surface horizons. Complementarily,^78^ demonstrated that microbial necromass, particularly fungal, accounts for up to 50% of SOC in global grasslands and is directly tied to the stabilization of mineral-associated organic matter (MAOM), the most persistent soil C pool. This provides an ecological framework to interpret our results, where NTF favored ligninolytic and mycorrhizal fungi with high potential for necromass formation. In addition,^79^ emphasized that microbial metabolites (enzymes, extracellular polysaccharides, siderophores) play a decisive role in soil aggregation and necromass formation, processes enhanced under NTF due to reduced disturbance and residue conservation.

The enrichment of *Glomeromycota* and *Aphelidiomycota* under NTF (Fig. 5b) highlights the role of symbiotic fungi in the formation of recalcitrant C. Global studies confirm that mycorrhizal associations are key drivers of C retention^69,80^, and their persistence is strongly shaped by management practices^81^. This suggests that conservation-oriented practices foster symbiotic communities that enhance soil resilience against C loss.

From a functional perspective, our results confirm the distinct contributions of bacteria and fungi to soil processes. Bacteria maintained redundant core functions such as C metabolism, glycolysis/gluconeogenesis, and amino acid biosynthesis (Fig. 7a), indicating functional resilience across both systems. In contrast, fungi exhibited system-specific guilds (Fig. 7b). NTF favored a diverse set of saprotrophic and ectomycorrhizal guilds, including multiple plant and wood saprotrophs, which are critical for the decomposition of complex residues, necromass formation, and the transfer of recalcitrant C into soil aggregates. Similar patterns have been reported globally, where ectomycorrhizal and saprotrophic fungi are recognized as key contributors to long-term SOC stabilization^82^. Reference^67^ further argues that conservation systems promote microbial traits and community configurations often associated with more conservative carbon cycling and greater incorporation of carbon into biomass and necromass, rather than rapid respiratory losses, thereby contributing to more stable SOC pools.

In contrast, CT enriched guilds such as Animal Pathogen–Fungal Parasite–Undefined Saprotroph, reflecting communities adapted to disturbed environments with nutrient fluctuations. These guilds are typically associated with opportunistic lifestyles that contribute less to long-term C storage and more to short-term nutrient pulses, reinforcing the idea that disturbance reduces the alignment of fungal functions with C stabilization^34,75^. Fungal functional ordination (Fig. S1) further supported this distinction: guilds under CT were structured primarily by soluble nutrient parameters (K_sol, Sulf_sol, N_NO₃) and EC, indicating that nutrient availability driven by disturbance shapes community function. In contrast, guilds under NTF were more strongly associated with SPR, suggesting that reduced tillage modifies soil physical resistance in ways that favor colonization strategies of guilds adapted to more stable environments^83^. Altogether, these results emphasize that fungal guilds act as mediators between soil structure, nutrient cycling, and long-term C sequestration, reinforcing the central role of fungi in linking conservation agriculture with climate change mitigation. This is consistent with evidence showing that land-use legacies shape microbial structure^12^.

Community assembly analysis revealed a non-random pattern dominated by environmental filtering (phylogenetic clustering) in both bacteria and fungi (Fig. 8). This is in line with niche theory^84^, where edaphic properties act as selective drivers, suggesting that NTF, by gradually modifying these conditions, steers communities toward functionally aligned states that favor C stabilization^85^. Similar conclusions were drawn by^86^, who showed that intensive management disrupts beneficial microbial groups and shifts community composition, reinforcing the idea that agricultural practices act as filters shaping microbial assembly and, consequently, soil C dynamics.

In general, our results show that NTF not only increases the surface SOC stock (Fig. 2a) but also reorganizes fungal communities toward groups with a greater capacity for C stabilization (Figs. 6 and 8), whereas CT promotes more opportunistic communities that are less oriented toward this function. These findings reinforce the notion that fungi are central mediators of the soil C cycle in agroecosystems, a role also highlighted in global studies linking the soil microbiome with climate change mitigation^11,87,88^. They are also consistent with evidence that conservation practices, by reducing physical disturbances and promoting more stable edaphic conditions, enhance the capacity of the microbiome to sustain ecosystem services^71,89^. At the regional scale,^90^ emphasize that grassland management in the Flooding Pampa directly conditions the balance between productivity and ecosystem services, including C sequestration, thus placing our results within a broader framework of agroecological sustainability.

In summary, this study shows that NTF modifies not only the physical and chemical properties of the soil but also the composition of microbial communities. Whereas bacterial communities maintained a more stable composition, fungi responded more sensitively to edaphic parameters and reduced disturbance, reorganizing into inferred guilds with greater potential to contribute to C stabilization. As noted by^67^, reduced-disturbance practices not only alter community structure but also decrease the temporal variability in CUE, consolidating more resilient systems that are less prone to rapid C losses. In our case, after more than two decades, NTF appears to have promoted a microbiome potentially oriented toward more conservative carbon cycling and stabilization, whereas CT favored opportunistic communities associated with faster mineralization dynamics. These findings reinforce that, even from a functionally inferred perspective, fungi emerge as key mediators linking soil structure, microbial composition, and C dynamics. As highlighted by^72^, management effects leave long-term microbial legacies; in our case, after more than two decades, NTF consolidated a fungal assemblage that stabilizes C, in agreement with global evidence from^78^ on the central role of necromass in grasslands. Therefore, conservation agriculture emerges as a cost-effective strategy for climate change mitigation^65^, comparable in relevance to nature-based solutions that prioritize restoration and plant biodiversity. These interpretations are based on functional inferences rather than direct physiological measurements, but they are consistent with the observed soil properties, community structure, and phylogenetic patterns.

## Conclusions

Our results highlight that long-term NTF promotes soil conditions and fungal community assemblages with a greater inferred potential to be associated with carbon stabilization processes, whereas CT favors opportunistic groups less aligned with long-term C sequestration. Although these findings are based on functional inferences rather than direct measurements, they are consistent with global trends and provide valuable evidence that conservation practices can enhance the role of the soil microbiome in climate change mitigation. This dual effect physical stabilization of SOM and functional reorganization of fungal guilds highlights the importance of incorporating microbiome-based indicators into sustainable agricultural policies in the Pampas region, directly supporting the UN’s SDG 13 (Climate Action).

## Data Availability

The raw 16S rRNA gene and ITS2 amplicon sequence data, along with their associated metadata, are publicly available in the European Nucleotide Archive (ENA) under accession number PRJEB97327.

## Declarations

### Permissions

The authors confirm that they had full permission to conduct the study at the Chascomús Integrated Experimental Farm (CEICh)(Buenos Aires, Argentina). Institutional authorization was provided by the Director, Matías Andrés Bailleres, co-author of this study.
